# In Envelope Additive/Subtractive Manufacturing and Thermal Post-Processing of Inconel 718

**DOI:** 10.3390/ma16010001

**Published:** 2022-12-20

**Authors:** Sila Ece Atabay, Priti Wanjara, Fabrice Bernier, Sheida Sarafan, Javad Gholipour, Josh Soost, Robert Amos, Prakash Patnaik, Mathieu Brochu

**Affiliations:** 1 National Research Council Canada, Transportation and Manufacturing Division, Montréal, QC H3T 1J4, Canada; 2 Matsuura Machinery USA Inc., St. Paul, MN 55102, USA; 3 Department of National Defence, Directorate of Technical Airworthiness and Engineering Support (DTAES), Ottawa, ON K1A 0K2, Canada; 4 Department of Mining and Materials Engineering, McGill University, Montréal, QC H3A 0C5, Canada

**Keywords:** additive/subtractive manufacturing, laser powder bed fusion, post-processing, heat treatment, Inconel 718, microstructure, mechanical properties, fractography

## Abstract

This study investigated the application of an in envelope additive/subtractive (LPBF) manufacturing method (Matsuura LUMEX-Avance-25) to fabricate IN718 benchmarking coupons. The coupons were then examined comprehensively for surface finish both with and without high-speed micro-machining. The microstructure of the manufactured IN718 coupons was investigated thoroughly in the as-fabricated condition and following three different standard and one non-standard post-processing heat treatments. As built coupons revealed columnar grain morphology mainly along the <100> direction with a cellular dendritic sub-grain structure and without any strengthening precipitates. Grain size, aspect ratio, and texture were maintained after each of the applied four heat treatments. Only one of the standard heat treatments resulted in the δ phase formation. The other three heat treatments effectively dissolved the Laves phase preventing the δ formation while promoting the formation of γ′/γ″ precipitates. Despite the observed differences in their microstructures, all of the heat treatments resulted in similar yield and ultimate tensile strength values that ranged between 1103–1205 MPa and 1347–1387 MPa, respectively. These values are above the minimum requirements of 1034 MPa and 1241 MPa for the wrought material. The non-standard heat treatment provided the highest elongation of 24.0 ± 0.1% amongst all the heat-treated specimens without a significant loss in strength, while the standard heat treatment for the wrought parts resulted in the lowest elongation of 18.3 ± 0.7% due to the presence of δ phase.

## 1. Introduction

Inconel^®^ alloy 718 (IN718) is a precipitation-hardenable nickel-based superalloy, comprised of metastable disc-shaped γ″ precipitates with a body centered tetragonal (BCT) structure and spherical or cuboidal γ′ precipitates with an ordered L12 structure, dispersed in a face centered cubic (FCC) γ matrix [1,2]. In addition to these phases, NbC and δ phases are observed in the IN718 microstructure [3,4]. Among these precipitates, γ″ and γ′ are the main strengthening precipitates providing superior elevated temperature mechanical properties, whereas the NbC and controlled precipitation of the δ phase contribute to the microstructural stability during high temperature exposure by providing resistance to grain boundary sliding [5]. Besides high strength, creep resistance, and fatigue strength at temperatures up to 700 °C, IN718 is also known to have reasonably good weldability due to its relatively sluggish precipitation kinetics [6]. This combination of properties has rendered IN718 as the material of choice for the hot section aero-engine structural components, as evidenced by this alloy making up more than 30% of the total weight of a modern aircraft engine [7,8].

IN718 is conventionally manufactured by a sequence of processing operations that include casting, hot working (i.e., by forging, rolling, and/or extrusion), heat treatment, and machining to the final geometry. However, this conventional manufacturing route provides very limited degrees of freedom for fabricating geometrically complex components [9]. Moreover, the same mechanical properties (e.g., high hardness, low thermal conductivity, high strength, etc.) that render IN718 attractive for high temperature applications also pose challenges for subtractive processing [10]. Thus, for complex geometries with high buy-to-fly ratios, alternate/sustainable manufacturing approaches to conventional processing of difficult-to-cut IN718 are especially critical to effectively reduce manufacturing costs and high-value material waste [4].

Laser powder bed fusion (LPBF) is one of the main additive manufacturing (AM) processes, which involves selective melting and solidification of a thin layer of powder with the aid of a predefined laser raster path [11]. LPBF offers design flexibility by enabling the fabrication of near-net-shape or net-shape components having complex geometries with reduced material waste [12,13]. However, LPBF AM still has certain drawbacks that require further investigations and optimization for specific geometries and components. A key concern for aerospace and defense applications—where fatigue-critical performance is important—is the poor surface quality of the LPBF fabricated parts that require post-process machining, which can obliterate the cost benefits associated with additive processing [14]. Hence, there is rising interest to use a hybrid manufacturing technique—involving a single (in envelope) setup for both additive and subtractive processing—for the production of complex geometry parts with high-quality surface finishes and tight dimensional tolerances [15,16]. Additionally, the extremely high heating and cooling rates involved during LPBF can suppress the precipitation of strengthening phases and lead to significantly finer solidification microstructures compared to the conventional manufacturing methods [17,18]. The repetitive heating and cooling cycles during AM also result in higher residual stresses in the final part [19,20]. Hence, post-processing heat treatments are required to relieve the residual stresses and to form the strengthening precipitates. However, since the as-built microstructure of the LPBF fabricated parts is significantly different compared to conventional production techniques, their microstructural response to the standard heat treatments shows differences [21,22]. Thus, studying post-process heat treatments for LPBF fabricated IN718 and their effect on the mechanical properties has significant importance for advancing the sustainable manufacturing of parts with superior and reliable/repeatable material performance, which is critical for high-end load-bearing applications in aerospace and defense.

Accordingly, this study investigated the application of an additive-subtractive hybrid (LPBF) manufacturing method (Matsuura LUMEX-Avance-25) to fabricate IN718 benchmarking coupons and examined their post-process heat treatment responses. Specifically, four different heat treatments were investigated: three based on standard practices for wrought IN718 and one non-standard process specially adapted for LPBF fabricated IN718. The coupons were first examined comprehensively for surface finish both with and without high-speed micro-machining. The microstructure of the additive/subtractive manufactured IN718 coupons was investigated thoroughly in the as-fabricated condition and following post-process heat treatment. Finally, hardness, tensile properties, and fracture behaviors were studied in both the as-fabricated and heat treated conditions.

## 2. Materials and Methods

**Figure 1:**
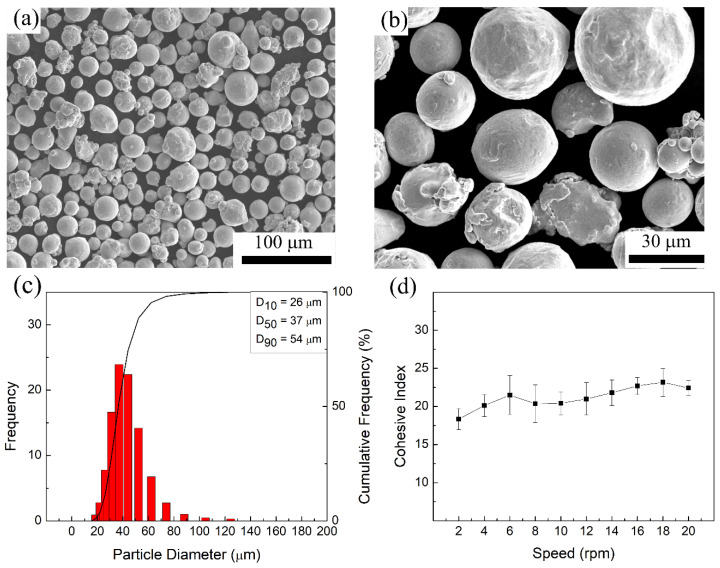
(**a**,**b**) Morphology, (**c**) particle size distribution, and (**d**) cohesive index of the starting IN718 powder.

The starting material used in this study was commercially available argon gas-atomized IN718 powder from Matsuura (St. Paul, MN, USA) with a nominal particle size of −45/+10 µm and an elemental composition as given in Table 1. The morphology of the IN718 powder particles was analyzed using a Hitachi SU3500 (Fukuoka, Japan) scanning electron microscope (SEM). As shown in Figure 1a,b; the majority of the as-received powder particles were spherical in shape with a few irregularities and satellites attached to their surfaces. The particle size distribution (PSD) of the IN718 powder was measured using an LA-920 Horiba (Kyoto, Japan) laser particle size analyzer and the results are shown in Figure 1c. The PSD analysis showed a normal distribution with D_10_, D_50_, and D_90_ values of 26 μm, 37 μm, and 54 μm, respectively. Flowability and the apparent density of the powder were assessed using Hall and Carney funnels according to the specifications in ASTM B213 [23] and ASTM B964 [24]. The measured respective flow rates of the IN718 powder from the Hall and Carney funnels were 25 ± 1 s and 4 ± 2 s for 50 mg of powder, indicating that the powder has suitable flowability to be used in the LPBF process. The apparent density of the powder was assessed as 3.92 g/cm^3^. In addition, a dynamic flowability analysis was conducted using a GranuDrum (Avans, Belgium) rotating drum instrument. Figure 1d shows the cohesive index (CI) of the IN718 powder as a function of the drum rotational speed from 2–30 rpm. The measured CI values, which ranged between 18–22, were statistically similar for the rotational speeds tested in this study. It is suggested that metal powders having CI values lower than 24 show good flowability and spreadability characteristics, resulting in a uniform powder layer for LPBF processing [25,26].

**Figure 2:**
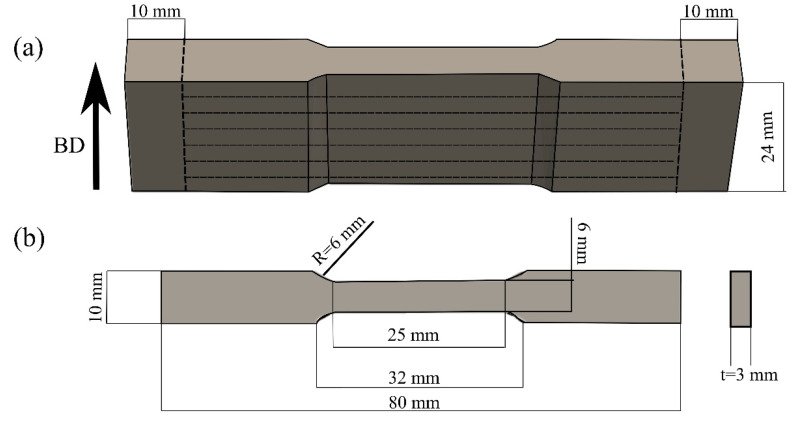
(**a**) Geometry of coupons fabricated by in envelope additive (LPBF)/subtractive manufacturing showing the outlines for extracting the metallographic sections and tensile specimens, and (**b**) standard sub-size tensile specimen geometry according to ASTM E8M-22 [28].

The coupon geometry represented in Figure 2a was additively manufactured with a Matsuura LUMEX Avance-25 hybrid LPBF and high-speed micro-milling system using a Yb fiber laser having a maximum power output of 400 W and a beam diameter at the focus of 200 μm. Four coupons were built under a protective nitrogen gas atmosphere (with less than 1% oxygen) on a precision ground 4140 steel baseplate maintained at 50 °C. It is noteworthy that the surface of the baseplate was a demagnetized (Electro-Matic model A13-1, R.B. Annis, ELMATCO, Chicago, IL, USA) to a magnetic field of <0.2 Gauss before LPBF processing. The four coupons were fabricated using a bidirectional scanning strategy with a 90° rotation between the layers. A laser power of 240 W, a hatch distance of 110 µm, and a layer thickness of 50 µm were used for the LPBF process. The laser scanning speed was set to 700 mm/s and 500 mm/s for the infill and contour scans, respectively. After the coupons were printed, milling was performed at a feed rate of 2000 mm/min on the profiles along the two side faces of the reduced cross-section, as well as on the final top surface of the entire part; 0.25 mm of material was removed from each surface.

The four coupons were removed from the baseplate using electro-discharge machining (EDM) (FANUC Robocut C400iB, Oshino-mura, Yamanashi, Japan) with a brass wire having a diameter of 0.2 mm. The surface quality of the coupons was evaluated by measuring the linear and areal roughness parameters on both as-built (AB) and machined faces. Linear roughness profiles were assessed using a portable Surftest SJ-210 (4 mN type profilometer, Mitutoyo Aurora, IL, USA) with a tip radius of 2 µm. The arithmetic mean height (R_a_), and maximum height (R_z_) values were derived from a primary line profile with a length of 6 mm by suppressing the long-wave component using a high-pass filter with a cut-off of λ_c_ = 0.8 mm. The areal roughness parameters, namely S_a_, and S_z,_ were measured by a 3D laser scanning confocal microscope (Keyence VK-X250, Osaka, Japan) on both surfaces, as per ISO 25178-2 [27].

Metallographic sections (10 × 10 × 24 mm^3^) and tensile specimens were then extracted from each of the four coupons, as shown in Figure 2a. The metallographic sections were used to extract representative specimens for heat treatment, density measurement, X-ray micro-CT inspection, microstructural analysis, phase analysis by X-ray diffraction, and microhardness testing. By contrast, the extracted tensile specimens (3 mm-thick) were used for tensile testing and conformed to the standard sub-size geometry in ASTM E8M-22 [28] with a gauge length of 25 mm and width of 6 mm as shown in Figure 2b.

The representative specimens for heat treatment (HT) were sectioned parallel to the building direction and subjected to four different thermal cycles, details of which are given in Table 2. For all five of the AB and heat-treated (HT) specimen conditions, the porosity distribution and pore size were evaluated using X-ray micro-CT inspection with a Nikon HMXST 225 system (Nikon Metrology Inc., USA) equipped with a Perkin-Elmer 1621AN CsI (2000 × 2000 pixels, 40 cm × 40 cm, 200 µm/pixel) detector panel. The scans were undertaken at a magnification level of 71X, which gave a voxel size of 2.84 µm. The X-ray micro-CT was operated at a voltage of 165 kV, a current of 50 µA with a 0.125-mm Ag filter, and an exposure time of 1415 ms using four frames per projection. For image analysis, Dragonfly software was utilized for 3D reconstruction to analyze the volume and size distribution of the pores using manual segmentation. Features inferior to six voxels were filtered out of the analysis. Furthermore, the bulk density of the specimens was measured using the Archimedes method with a theoretical density value of 8.19 g/cm^3^ to calculate the relative density of the AM IN718 alloy [29].

Residual stress measurements were conducted via X-ray diffraction (XRD) (cosα method) using a Pulstec μ-X360s portable X-ray residual stress analyzer. Measurements were conducted from the surfaces parallel and perpendicular to the recoating direction both along and perpendicular to the building direction.

Next, for microscopic examination, all five of the AB and HT specimens were sectioned, mounted, and ground planar using 600-grit SiC paper followed by polishing with 9 µm, 3 µm, and 1 µm diamond suspensions on a rigid composite disc followed by synthetic polishing cloths. Final polishing was performed using a 0.05 μm colloidal silica suspension on a Vibromet 2 polisher (Buehler, Longueuil, QC, Canada). Electron backscatter diffraction (EBSD) was applied to examine the grain morphology and texture for each specimen condition using a Hitachi SU3500 SEM under operating conditions of 15 kV with a 2 μm step size. The raw EBSD data were collected using Aztec data acquisition software and analyzed with HKL Channel 5 data processing software (Oxford Instruments NanoAnalysis, Concord, MA, USA). Pole figures of the {100}, {110}, and {111} planes were extracted from the EBSD data using a half-width of 10° and a cluster size of 5°. In addition, the grain diameter and aspect ratio of the grains were extracted using the inverse pole figure (IPF) maps obtained from the EBSD analysis. Furthermore, the phase analysis for each specimen was conducted by XRD analysis using a Bruker D8 Discovery X-Ray diffractometer with Co Kα1 radiation (wavelength 1.78897 Å) and operating at 35 kV with 45 mA.

To reveal the microstructural features of the IN718 alloy in the various specimen conditions, electro-etching was performed in a solution consisting of 12 mL H_3_PO_4_, 40 mL HNO_3_, and 48 mL H_2_SO_4_ at 6 V for 15 s [34]. A Keyence VK-X250 3D laser scanning confocal microscope was used to construct the 3D macrostructures. A JCM-7000 NeoScope™ benchtop scanning electron microscope (SEM) (JEOL, Akishima, Tokyo, Japan) equipped with an energy dispersive spectrometer (EDS) facilitated observation and characterization of the general microstructure at low magnifications and compositional analysis. A Hitachi SU8000 STEM (Fukuoka, Japan) equipped with an EDS was used for high magnification observation of the fine precipitates in the microstructure of the IN718 alloy. Five micrographs were collected from different regions of each specimen for image analysis using Image J software to determine the size of the precipitates.

Microhardness measurements were performed on the specimens using a Struers DuraScan 80 hardness tester (Ballerup, Denmark). Individual hardness indentations were measured on polished surfaces using a spacing of 0.5 mm and a 300 g load for 15 s to determine the hardness distribution in the IN718 alloy subjected to the various specimen conditions.

The room temperature tensile properties of the AB and HT specimens were determined using a 250-kN MTS load frame integrated with a laser extensometer and a non-contact optical 3D deformation measurement system (often referred to as digital image correlation (DIC)), Aramis^®^ GOM-Trillion Quality Systems, (King of Prussia, PA, USA). For each condition, three flat dog-bone tensile specimens with a geometry given in Figure 2b were tested. Before tensile testing, two pieces of retro-reflective tape were attached to one side of the tensile specimen to distinguish the gage section for the laser extensometer during testing. On the opposite side, the surface of the tensile specimen was first painted with a white background and then a high-contrast random pattern of black speckles was applied. As the functionality of the Aramis^®^ system is sensitive to the quality of this speckle pattern, verification of pattern recognition was performed before tensile testing to ensure proper strain recording along the entire gage length, as described in [35,36]. Tensile tests were conducted with a strain rate of 0.005 mm/mm/min up to yield and 0.05 mm/mm/min until fracture. To obtain the stress-strain curves and the related mechanical properties—yield strength (YS), ultimate tensile strength (UTS), and percent elongation—of the AB and HT specimens, the load data, collected by the tensile testing machine, were used to calculate the engineering stress, while the elongation was evaluated from the data collected by the extensometer. In addition, the strain maps captured by the Aramis^®^ system were used to assess the local deformation behavior of the specimens during tensile testing. Fractography analyses were conducted on the tested specimens after tensile fracture using a JCM-7000 NeoScope™ Benchtop SEM.

## 3. Results and Discussion

### 3.1. Surface Roughness

**Figure 3:**
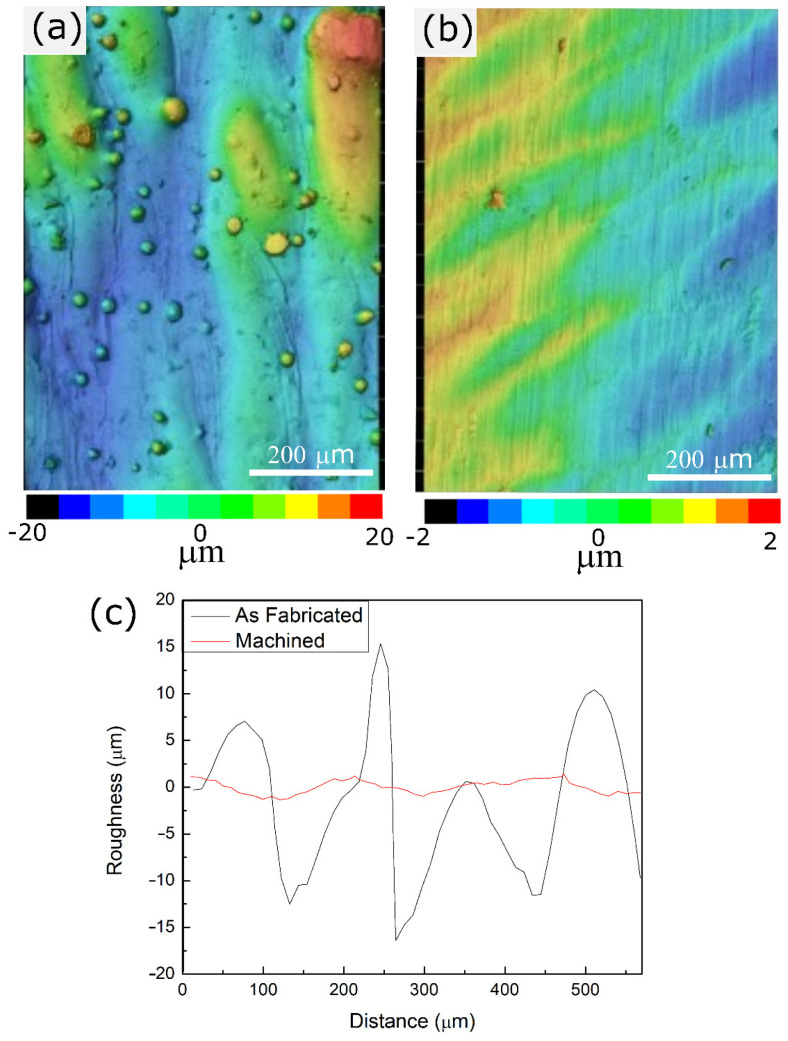
Surface topography characterization: (**a**) 2D surface profile of an as-built surface, (**b**) 2D surface profile of a machined surface, and (**c**) comparison of surface line profiles of as-built and machined surfaces.

The topography of the AB and machined side wall surfaces of the coupons parallel to the building direction was compared using a 3D laser scanning confocal microscope. Representative topological profiles of AB and machined surfaces are shown in Figure 3a,b, respectively. The AB surface consists of periodic perturbations along the building direction, forming as a result of the layer-by-layer processing involved in LPBF. Additionally, unmelted powder particles attached to the surface are clearly visible in Figure 3a. The representative line profiles of both surfaces are also compared in Figure 3c. Both topological profiles and the height versus distance graph reveal that the surface quality of the AB surface is significantly lower than the machined surface. The variation in the height profile along the building direction of the AB surface is an order of magnitude higher compared to the machined surface. This can be attributed to the step-like perturbations and the unmelted powder particles observed on the AB surface.

Linear and areal roughness values for both the AB and machined surfaces were also measured and are summarized in Table 3. Linear roughness parameters of R_a_ and R_z_ of the AB surfaces were 5.24 µm and 30.94 µm, respectively. After machining, approximately 90% of reduction was observed in both parameters to 0.66 µm and 4.05 µm. Areal roughness parameters also revealed a similar trend. Surface roughness parameters S_a_ and S_z_ were measured as 9.17 µm and 66.52 µm, respectively, for the AB surface. These values decreased by 80–85% to 1.43 µm and 13.90 µm after machining.

It has been shown that the surface roughness of LPBF fabricated parts is directly affected by the process parameters [37]. Balbaa et al. [38] observed the R_a_ value of 12 µm for LPBF fabricated IN718, and this value could be decreased to 2.1 µm when the scan speed and the hatch spacing were reduced. Kladovasilakis et al. [39] also suggested that it is possible to reduce the roughness with a reduction in the scan speed. However, implementation of the suggested parameter modifications to improve the surface quality limits the process parameter window and results in an increase in the processing time. During additive/subtractive manufacturing, the LPBF process parameters can be adjusted for high-efficiency production without consideration of the surface roughness. Therefore, the in envelope milling process not only provides a superior surface finish but also greater flexibility to optimize the LPBF process parameters for high efficiency.

### 3.2. Density

**Figure 4:**
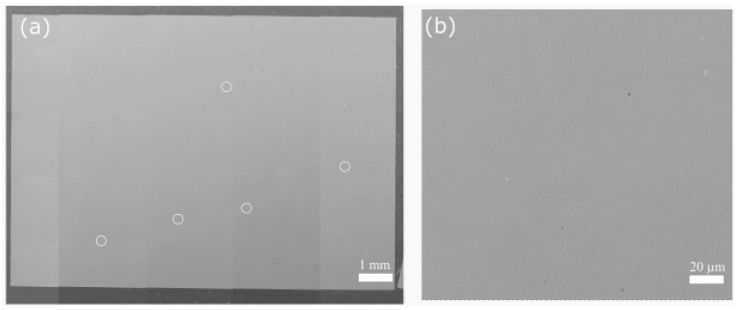
As-polished SEM micrograph of (**a**) the cross-section of the AB specimen along with (**b**) a representative higher magnification image. The white circles in (**a**) demarcate small gas pores in the AB IN718.

Figure 4a shows a montage of SEM micrographs taken from a representative as-polished cross-section of the AB specimen. The AB specimen was fully dense without the visible presence of defects such as cracks, lack of fusion, or keyhole porosity. The only visible defect on the specimen cross-section was small gas porosity highlighted using white circles in Figure 4a. The higher magnification SEM micrograph in Figure 4b more clearly demonstrates the characteristics of the small gas pores present in the microstructure and the absence of any other defects.

The bulk densities of the AB specimens were measured using the Archimedes method and µCT analysis for each condition, and the results are shown in Table 4. The density of the specimens was statistically similar to each other. Average Archimedean density was measured as 8.16 g/cm^3^ in the AB condition and 8.18 g/cm^3^ after all the applied heat treatments. These values correspond to a relative density of 99.6% and 99.9%, respectively. The µ-CT analysis revealed that the total pore volume in the analyzed volume (approximately 24 mm^3^) of the samples varied between 0.003 to 0.008%. Hence, the relative density of the specimens calculated from the µ-CT analysis was 99.9% similar to the Archimedes method. Additionally, the number of pores per volume was calculated for each specimen condition. As shown in Table 4, the pore density values for each specimen condition were very close to each other and ranged between 24 and 37, further supporting the findings of similar densities.

**Figure 5:**
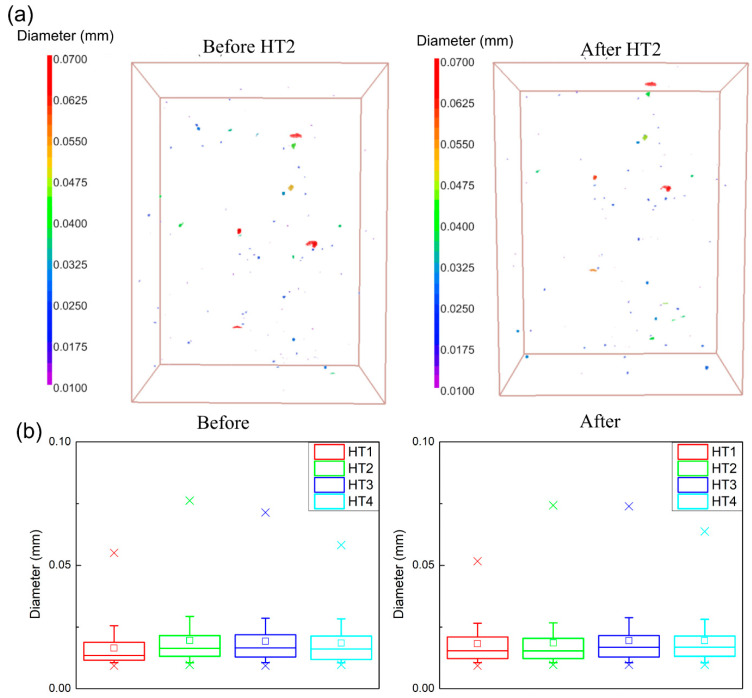
(**a**) Representative 3D visualization of the μ-CT analysis, (**b**) pore size distribution for each specimen condition before and after the applied heat treatments.

Figure 5a shows the 3D pore distribution of the HT2 specimen before and after the heat treatment in order to visualize and quantify the void distribution. As depicted by the µ-CT analysis, the pores had no preferred locations and heat treatment did not have a significant effect on the pore size and distribution. Small and spherical gas porosities—with an average maximum feret (D_feretmax_) smaller than 50 µm—along with a small number of large pores—with a D_feretmax_ of 70 µm—were observed both before and after the applied heat treatment. Only one representative specimen condition has been presented in Figure 5 for visualization since all the other specimens had similar results. In order to further analyze and compare the pore morphology and its possible effects on the mechanical properties, the pore size distribution of each specimen condition was calculated using the data obtained from the µ-CT analysis and the results are shown as box graphs in Figure 5b. The ‘x’ markers represent the 1st and 99th percentile in this graph, whereas the 10th and 90th percentiles are represented by the outliers. Additionally, each line of the boxes represents the 25th, 50th, and 75th percentiles, respectively, from the bottom to the top. The average pore size is represented by the small square marker inside the boxes. The first graph shows the pore size distribution before each of the applied heat treatments; after heat treatment, another µ-CT scan was conducted in the same region of each specimen. In all the analyzed specimens, 90% of the pores had a D_feretmax_ smaller than 30 µm, both before and after the heat treatments. It is worth noting that specimens collected from the four different regions of the AB coupons showed statistically similar pore size distributions, which points to the homogeneity within the coupons. Additionally, it is clearly seen that the size distribution range, mean and average size values of the pores were almost identical before and after each of the applied heat treatments. Hence, it can be concluded that none of the applied heat treatments had any effect on the porosity of the additive(LPBF)/subtractive manufactured IN718 specimens.

### 3.3. Microstructural Characterization

Surface residual stress measurements were conducted prior to microstructural analysis. The measured surface residual stresses from the machined surfaces ranged between −330 ± 90 MPa and −859 ± 50 MPa. The maximum residual stresses observed were on the surface parallel to the recoating direction and along the building direction. Despite the high compressive surface residual stresses, no variation in the grain morphology and microstructure could be detected due to these residual stresses. Hence, its effect is neglected for the rest of the analysis in this study.

#### 3.3.1. Grain Morphology and Texture

**Figure 6:**
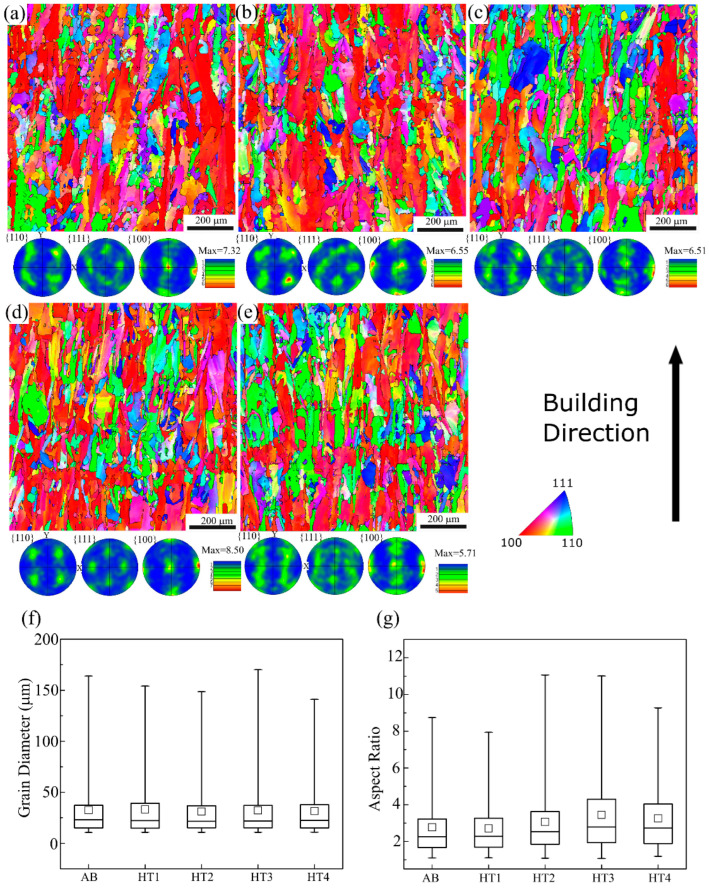
IPF orientation maps and corresponding pole figures of the (**a**) AB, (**b**) HT1, (**c**) HT2, (**d**) HT3, and (**e**) HT4 specimens. Box plots show the distributions of (**f**) grain diameter, and (**g**) aspect ratio for each specimen condition.

An EBSD analysis was conducted to assess the grain morphology and crystallographic texture in the AB and heat-treated conditions. IPF orientation maps along with the corresponding pole figures for each specimen condition are shown in Figure 6. The solid black lines in the orientation maps represent the high angle grain boundaries for misorientations larger than 15°. As depicted in Figure 6a, the AB specimen mainly consists of columnar grains elongated parallel to the build direction. The long axis of the columnar grains is predominantly orientated along the <100> direction, which is the preferred growth orientation in FCC materials [21,40]. The corresponding pole figures reveal that the texture of the AB specimen has a mixture of cube and Goss components with intensities represented by the multiples of uniform density (MUD) that had a maximum value of 7.32. This type of grain morphology is commonly observed during LPBF processing of IN718 and other Ni-based superalloys due to the directional heat transfer and epitaxial growth of the solidifying grains across several layers [34,41,42].

Figure 6b–e show the IPF orientation maps after the specimens were subjected to HT1, HT2, HT3, and HT4, respectively. As clearly noticed in these representative images, none of the applied heat treatments altered the columnar grain morphology oriented mainly along the <100> direction observed in the AB condition. Similarly, all the specimens revealed a mixture of Goss and cube texture components with peak intensity values very close to each other. The highest and lowest MUD values of 8.50 and 5.71 were observed for the HT3 and HT4 conditions, respectively.

Furthermore, the diameter and the aspect ratio of the grains were calculated using the IPF orientation maps and the obtained results were compared for each specimen condition. The line intercept method in ASTM E1382-97 was utilized to measure the grain diameter. The aspect ratio corresponds to the length-to-width ratio of the fitted ellipses over the grains. Hence, an aspect ratio value of 1 corresponds to an equiaxed structure, and the columnarity increases as this value increases. The grain size distribution of each condition is shown as a box graph in Figure 6f. Two ends of the vertical lines in the graph represent the 1st and 99th percentiles, whereas three horizontal lines within the large rectangle represent the 25th, 50th, and 75th percentiles from the bottom to the top. The average grain size is shown with the small squares. As depicted in the box graph, both the mean and the average grain size values are very similar to each other for all five specimen conditions. This shows that the solidification grain morphology of the in envelope additive/subtractive manufactured IN718 remained unaffected after the applied heat treatments. Hence no recrystallization and/or grain growth of the grains occurred during the post-process heat treatments. Additionally, the distribution of the aspect ratio for each condition was statistically similar, as shown in Figure 6g. Even though the mean and average of the aspect ratio values are very close for each specimen, the 75th and 99th percentile values are the highest for the HT2 and HT3 specimens. These two heat treatment cycles include a homogenization step at a higher temperature compared to the others. Hence, it is possible that the coalescence of grains occurred during this step, thereby resulting in the high aspect ratio values observed in these two specimens.

#### 3.3.2. Phase Identification

**Figure 7:**
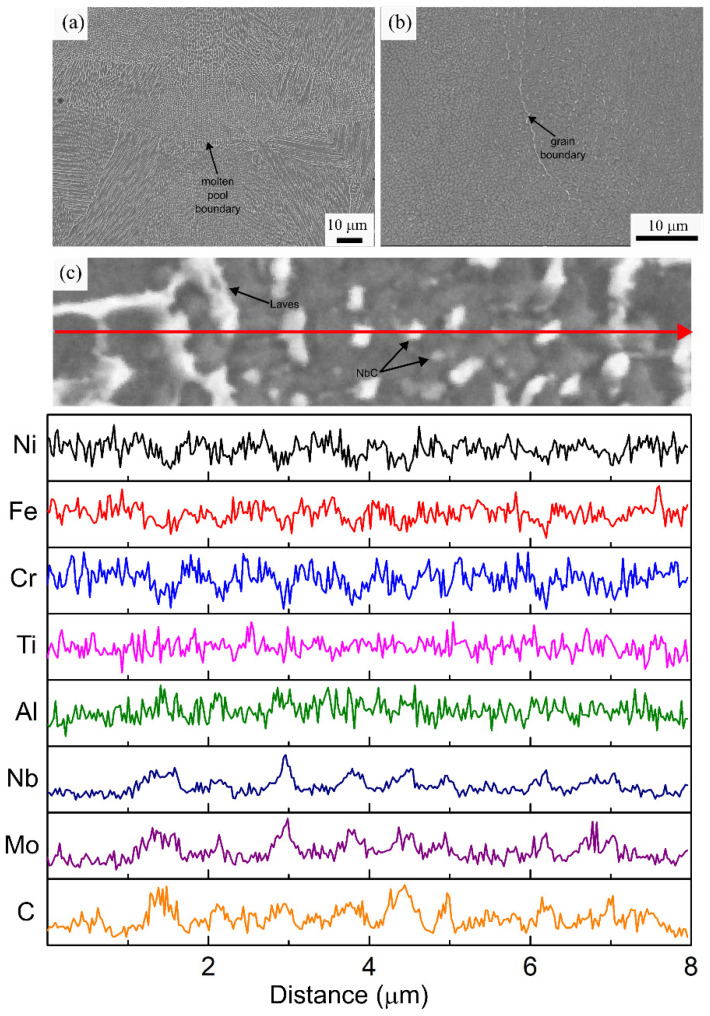
(**a**,**b**) Representative SEM micrographs, (**c**) EDS line scan analysis of the AB specimen along the marked red arrow.

A detailed microstructural analysis was conducted for each specimen condition using a combination of SEM, EDS, and XRD analysis. The low magnification SEM micrograph of the AB specimen reveals the presence of molten pool boundaries and columnar grains elongated over multiple molten pools, as shown in Figure 7a. Additionally, a fine cellular dendritic sub-grain structure is observed. The primary dendrite arms have grown from the bottom of the molten pool parallel to the building direction, which is the direction of the highest thermal gradient. According to the theory of alloy solidification, the morphology and size of the microstructural features are determined by two factors, namely the thermal gradient (G) and solidification front velocity (R). As the G/R ratio changes from high to low values, planar, cellular, columnar dendritic, and equiaxed dendritic microstructures will be observed. The large thermal gradients (10^5^–10^6^ K/s) observed in LPBF promotes columnar grain formation [43,44,45]. The size of the microstructural features is associated with the cooling rate G*R. The cooling rate during solidification can be calculated using the primary dendrite arm spacing (PDAS) according to the equation: PDAS = αε^−b^ (α = 50 μm (K/s) and b = 1/3 for Ni-based superalloys) [46,47]. The average PDAS was measured as 0.697 ± 0.200 µm, which corresponds to an average cooling rate of 3.69 × 10^5^ K/s during the processing of the IN718 alloy. Similar PDAS and cooling rate values were reported for LPBF processing of IN718 using various stand-alone machines [22,48]. Hence, the processing conditions used in this work, which are optimized for an improved production rate, resulted in a similar solidification microstructure observed in the stand-alone LPBF machines. Precipitation of γ′ and γ″ was not observed in the AB specimen. This can be associated with extremely high cooling rates, which inhibit precipitation during the solidification and subsequent heating and cooling cycles. The presence of bright interdendritic phases was observed in the higher magnification image in Figure 7b. The high-temperature gradients and cooling rates observed in the LPBF process amplify the compositional fluctuations ahead of the solidification interface and promote the formation of these secondary phases due to segregation. These phases are enriched in Nb, and Mo and depleted in Ni, Fe, and Cr according to the EDS line scan analysis displayed in Figure 7c. Elemental segregation during solidification is determined by the partitioning coefficient of the alloying elements. Elements with partition coefficients close to unity remain in solution with the solid, whereas elements with lower partition coefficients, such as Nb, Ti, and Mo, are segregated in the liquid [49]. Once a local chemical threshold is reached, the formation of NbC particles and Laves phase, which are indicated using black arrows, is observed in these intercellular regions. The average size of the carbide particles and the Laves phase was measured as 59.2 ± 24.3 nm and 212.6 ± 57.4 nm, respectively. These phases are commonly reported in the AB microstructure of LPBF fabricated IN718 [2,21,22]. The presence of these fine, discrete carbide particles pins the grain boundaries and provides microstructural stability during elevated temperatures. However, the Laves phase is known to be detrimental to the mechanical properties. Specifically, as Nb is a major constituent of the strengthening precipitates γ′ and γ″; formation of the Laves phase results in depletion of the available Nb, resulting in inhibition of the precipitation and reduction in strength [22,47]. Hence, dissolution of this phase through homogenization and/or solutionizing heat treatments is crucial to improving the mechanical properties of IN718 through precipitation hardening.

**Figure 8:**
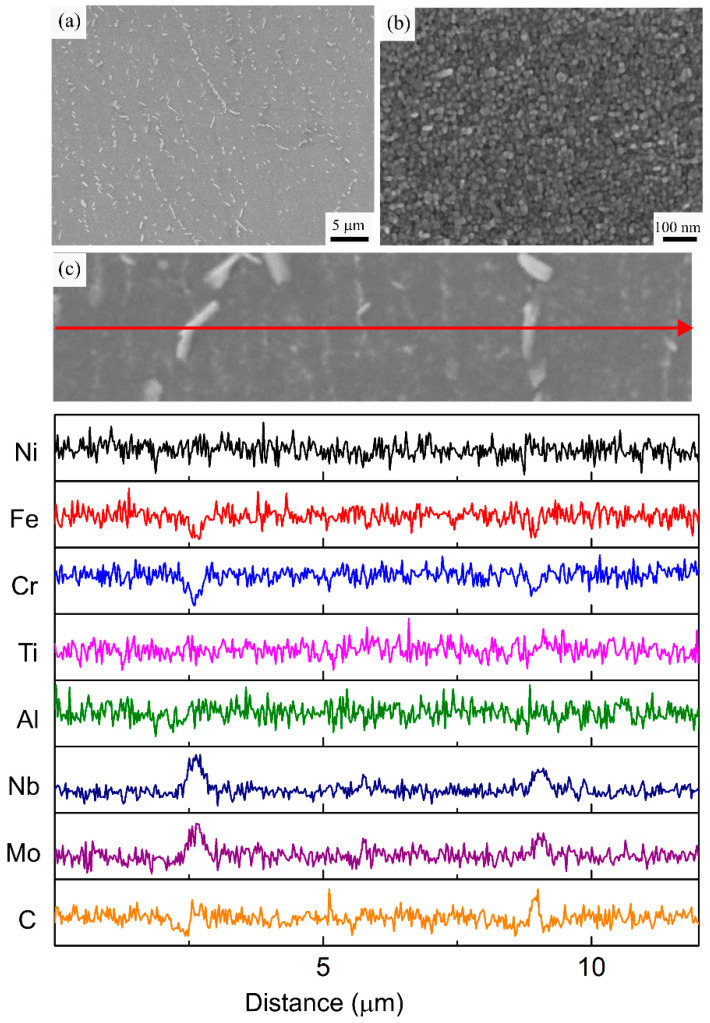
(**a**,**b**) Representative SEM micrographs, (**c**) EDS line scan analysis of the HT1 specimen along the marked red arrow.

Upon standard solutionizing and aging heat treatment (HT1) designed for the wrought IN718 parts, dissolution of the molten pool boundaries was observed in the HT1 specimen. Additionally, Figure 8a reveals precipitation of the needle-like δ phase at the intercellular area and grain boundaries. The average size of these needle-like particles along their minor and major axes was measured as 195.4 ± 93.5 nm and 900.6 ± 314.0 nm, respectively. During solutionizing at 980 °C, dissolution of the Laves phase starts, releasing large amounts of Nb into the interdendritic area and resulting in the formation of the large needle-like δ phase at the cell and grain boundaries. The EDS line-scan analysis in Figure 8c shows that the δ needles are enriched in Nb and Mo and depleted in Fe and Cr. The elemental analysis indicates that the solution temperature was not high enough for Nb and Mo to go into the solution after the dissolution of the Laves phase. Since the solutionizing temperature is lower than the solvus temperature of the δ phase (≈ 1010 °C) the formation of the δ phase directly from the γ matrix was observed. Rapid formation and growth of the δ phase are frequently reported for LPBF fabricated IN718 after a solutionizing heat treatment at 980 °C [2,33,48,50]. Studies report that moderate amounts of δ phase contribute to high-temperature properties by providing grain boundary pinning. The unchanged grain morphology during the applied HT1 can be associated with this effect. However, this phase is also associated with a reduction in fracture toughness, high-temperature ductility, fatigue life, and corrosion resistance in intergranular zones due to its acicular shape [5,43]. Furthermore, the presence of large amounts of δ will deplete the γ matrix in terms of Nb and inhibit the formation of the main strengthening precipitates.

The high magnification SEM micrograph in Figure 8b shows that the HT1 also resulted in the precipitation of a mixture of fine γ′ and γ″ precipitates in the sub-grain structure. The resolution of the SEM was not significant enough to differentiate the two different types of precipitates. The size distribution of the precipitates was also analyzed using the linear intercept method. The size of the precipitates ranged between 12–30 nm with an average of 19 ± 4 nm. A similar precipitate size distribution was reported by Gallmeyer et al. [33] for LPBF fabricated IN718 subjected to HT1. The unimodal size distribution of the precipitates also confirms the absence of precipitation in the AB specimen.

**Figure 9:**
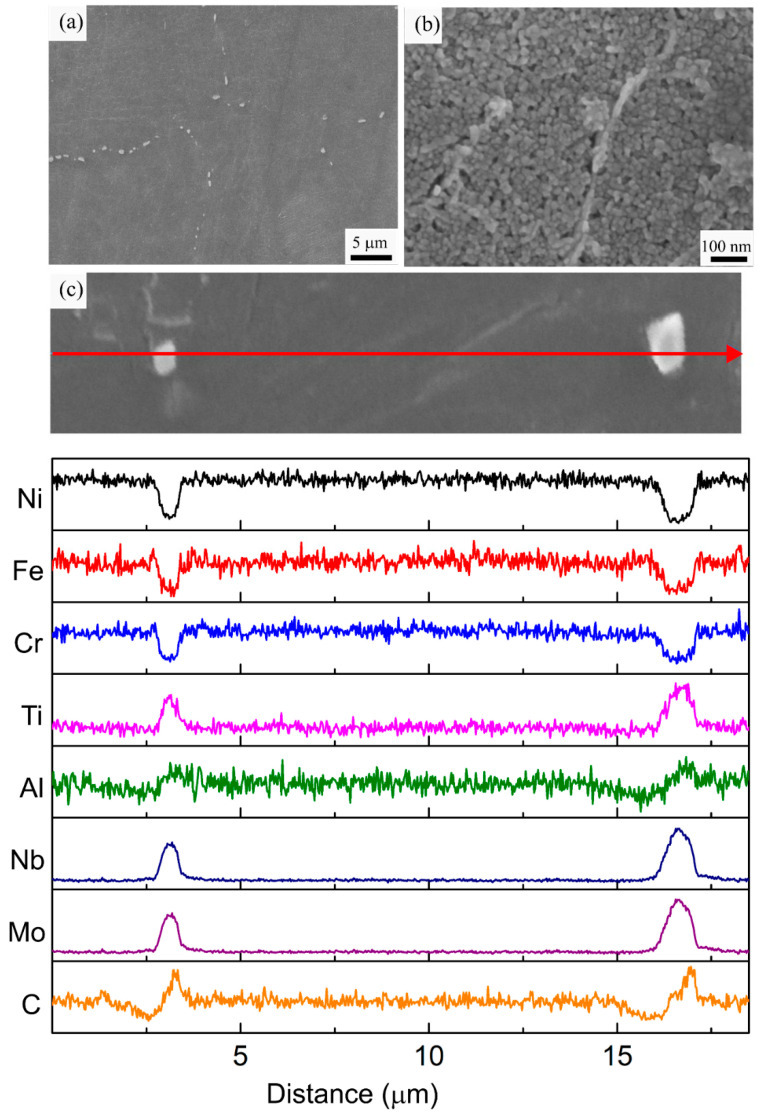
(**a**,**b**) Representative SEM micrographs, (**c**) EDS line scan analysis of the HT2 specimen along the marked red arrow.

The microstructural investigation of the HT2 specimen revealed that a homogenization heat treatment at a higher temperature (1080 °C) prevents δ phase formation. As depicted in Figure 9a, the molten pool boundaries and Laves phase observed in the AB specimen are completely dissolved after HT2. Additionally, the presence of large discrete blocky carbide particles with an average diameter of 412.2 ± 144.1 nm was observed along the grain boundaries. Similar to the MC carbides observed in the AB specimen, these carbide particles are also enriched in Nb, Mo, and Ti and lean in Ni, Fe, and Cr, as revealed by the EDS line scan analysis in Figure 9c. The homogenization temperature used in HT2 was above the solvus temperatures of the Laves and δ phases and below the solvus temperature of the MC carbides [5]. Therefore, in the HT2 specimen, the Laves phase dissolved during the homogenization step and the Nb released during its dissolution dissipated into the γ matrix instead of forming the δ needles observed in the HT1 specimen. The higher homogenization temperature in HT2 accelerated the diffusion and homogenization of the segregated elements. However, the MC carbides remained present in the microstructure and coarsened into blocky particles during the applied HT schedule. Nonetheless, the presence of the carbide particles on the grain boundaries provided grain boundary pinning, resulting in an unchanged grain morphology for the HT2 specimen. After homogenization, the same solutionizing step applied to the HT1 specimen was utilized but it did not result in δ formation, as all the available Nb was either in solution with the γ matrix or with the carbides. Hence, to prevent the formation of the hard and brittle δ phase, a higher temperature homogenization heat treatment is necessary.

Figure 9b shows that the two-step aging heat treatment resulted in a similar formation of fine strengthening γ′ and γ″ precipitates within the cellular microstructure in the HT2 specimen. The size of these precipitates ranged between 12–30 nm with an average of 21 ± 5 nm. The observed size distribution was very similar to the one observed in the HT1 specimen. Therefore, it can be remarked from the HT1 and HT2 specimens that applying the two-step aging treatment results in a similar precipitate size distribution, regardless of the presence of the δ phase.

**Figure 10:**
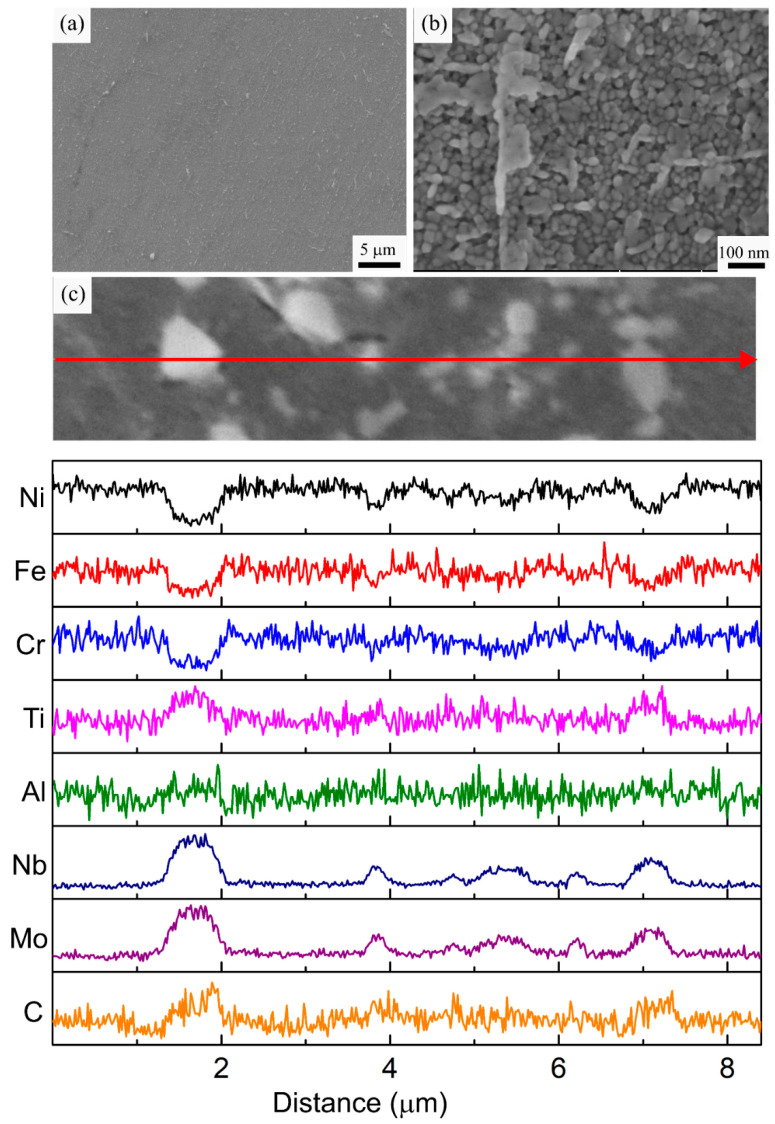
(**a**,**b**) Representative SEM micrographs, (**c**) EDS line scan analysis of the HT3 specimen along the marked red arrow.

The HT3 specimen was homogenized at a slightly lower temperature (1065 °C) than HT2 (1080 °C), followed by a similar two-step aging treatment. Even though homogenization was conducted at a lower temperature and for a shorter time for HT3 (relative to HT2), dissolution of the molten pool boundaries and the Laves phase occurred without δ phase precipitation in the HT3 specimen, similar to the HT2 specimen. Furthermore, Figure 10a reveals that blocky MC carbides are also present at the grain boundaries due to coarsening during the applied HT. The average diameter of these carbide particles was 307.8 ± 172.5 nm. The slower diffusion—at the lower temperature and shorter holding time—limited carbide coarsening in the HT3 specimen with a smaller average particle size (by 25%) compared to the HT2 specimen. In addition, the extra solutionizing step (980 °C for 1 h) applied to the HT2 specimen was removed for the HT3 specimen, which also contributed to the prevention of carbide coarsening in HT3. Elemental analysis of the carbides in the HT3 specimen is given in Figure 10c. The enrichment of Nb, Mo, and Ti is again clearly visible along with the depletion of Ni, Fe, and Cr, proving that these carbide particles are formed by the coarsening of the MC carbides observed in the AB specimen. Hence, after homogenization, a solutionizing step does not have a significant effect on the secondary phases observed in the final microstructure; it only causes the further coarsening of the undissolved carbides.

The higher magnification microstructural analysis of HT3 in Figure 10b shows the presence of strengthening precipitates within the cellular sub-grain structure similar to HT1 and HT2. However, the precipitates are coarser in the HT3 specimen with an average size of 33 ± 9 nm and a size distribution ranging between 18 to 58 nm. This difference can be attributed to both the difference in the aging conditions and the size of the carbides. Specifically, the temperatures used for both aging steps in HT3 were 30 °C higher relative to HT1 and HT2, which would promote a higher diffusion rate and precipitate coarsening in the HT3 specimen. The growth of the precipitates is also limited by the availability of Nb and Ti, as they are the primary alloying elements for the γ′ and γ″ phases. The smaller size of the carbides means that a lower amount of the available Nb and Ti is consumed by the carbides; hence the growth of the γ′ and γ″ precipitates is less limited by their availability.

**Figure 11:**
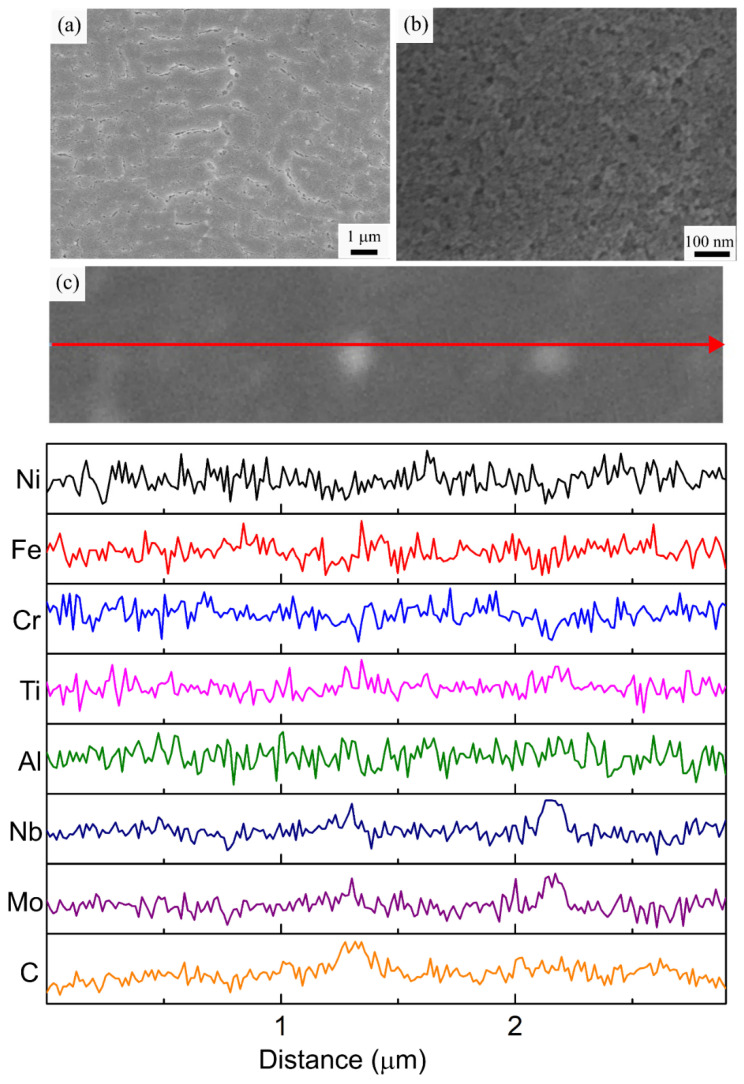
(**a**,**b**) Representative SEM micrographs, (**c**) EDS line scan analysis of the HT4 specimen along the marked red arrow.

It has been reported that the heat treatment response of LPBF fabricated IN718 shows significant differences compared to its wrought or cast counterparts [25]. Due to the unique fine dendritic structure of LPBF IN718, the required solutionizing time is shorter [42]. Hence, a nonstandard treatment (HT4) consisting of a high temperature solutionizing step (1020 °C) for a shorter time (¼ h) followed by a single-step aging treatment, as suggested by Gallmeyer et. al [25], was studied for the in envelope additive/subtractive manufactured IN718 specimens. Figure 11a shows that the cellular dendritic subgrain structure in the AB specimen is preserved in HT4, while the Laves phase has dissolved. This shows that the solutionizing conditions in HT4 (1020 °C, ¼ h) are sufficient to dissolve the Laves phase without any δ phase precipitation. Fine, discrete carbide particles along the cell and grain boundaries are also visible in the microstructure after HT4. The average diameter of these carbides was 84.4 ± 20.3 nm, which is just slightly larger than the carbides observed in the AB specimen, indicating limited carbide coarsening in the HT4 specimen. The pinning effect exerted by these fine carbide particles enabled the sub-grain and grain structure of the AB specimen to be maintained in the HT4 specimen. Similar to the carbide particles observed in the other specimens, these fine carbides were also rich in Nb, Mo, and Ti, as revealed by the EDS line scan analysis (Figure 11c).

Extremely fine strengthening precipitates are clearly visible in the high magnification SEM micrograph in Figure 11b. HT4 resulted in a finer size distribution of the strengthening precipitates compared to the other heat treatments. The γ′ and γ″ precipitates cannot be differentiated under the SEM, but the size range of the precipitate mixture was 8 to 21 nm, and the average size was 13 ± 3 nm, which is the smallest size distribution among all the specimens. γ″, γ′, and, γ′/γ″ coprecipitate sizes of 8 ± 2, 23 ± 4, and 18 ± 3 nm were reported previously using this same heat treatment on LPBF IN718 produced using a stand-alone machine with different process parameters [25]. The similar size range of the precipitates observed in the present study for additive (LPBF)/subtractive manufactured IN718 points to the robustness of HT4.

**Figure 12:**
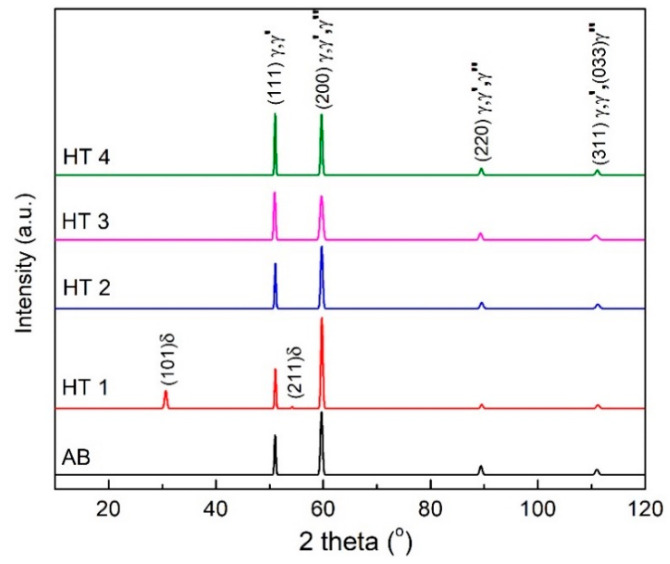
XRD diffraction patterns of the IN718 specimens in AB and HT conditions.

The XRD diffractograms of all the specimen conditions given in Figure 12 support the above microstructural analysis. All the specimens have similar XRD patterns except HT1. The peaks of γ, γ′, and γ″ cannot be differentiated by the XRD analysis, since there is an overlap between their γ(111)/γ′(111), γ(200)/γ′(200)/γ″(200), γ(220)/γ′(220)/γ″(220), and γ(311)/γ′(311)/γ″(033) peaks. Furthermore, no obvious peaks for carbides or the Laves phase were observed in the diffractograms. This could be attributed to their small size and low volume fraction. Two new peaks for the Ni_3_Nb orthorhombic δ phase appeared in the diffraction pattern of the HT1 specimen, confirming the presence of large amounts of the δ phase from this heat treatment. Similar to the observations from the above microstructural characterization, the δ phase was not present in the diffractogram of any other specimen except HT1.

### 3.4. Mechanical Characterization

#### 3.4.1. Hardness

**Figure 13:**
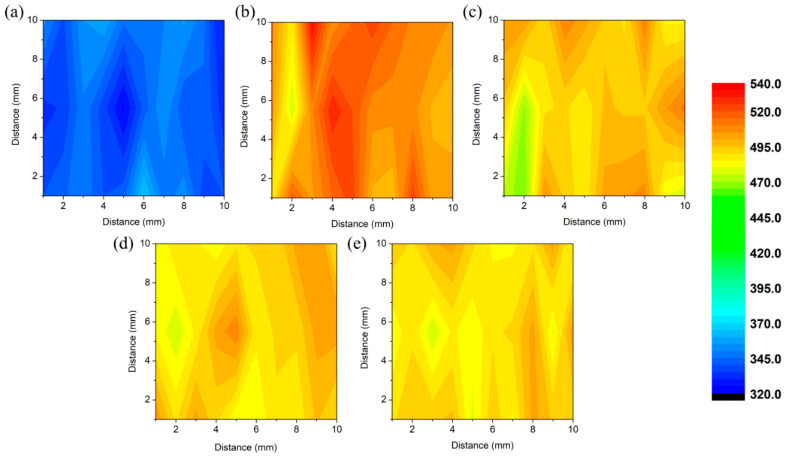
Microhardness maps of IN718 specimens in (**a**) AB, (**b**) HT1, (**c**) HT2, (**d**) HT3, and (**e**) HT4 conditions.

Systematic microhardness measurements were conducted from a representative 10 × 10 mm^2^ cross-section, and Vickers microhardness maps were constructed to understand the variation within the specimens and the effect of the applied heat treatments. As shown in the maps in Figure 13, the microhardness of all the specimens was homogeneous throughout the investigated cross-sections, proving that there were no significant local variations in the microstructure. The lowest average microhardness value of 339 ± 10 HV was calculated for the AB specimen. This could be associated with the absence of the strengthening precipitates in the AB condition. Similar microhardness values between 270 and 397 HV were reported previously for IN718 alloy fabricated by stand-alone LBPF technologies [51].

After the specimens were subjected to the four different heat treatments, hardness was increased by 44 to 49% due to the formation of strengthening precipitates and secondary phases. The average hardness value calculated for HT1, HT2, HT3, and HT4 specimens were 508 ± 13 HV, 493 ± 12 HV, 491 ± 9 HV, and 490 ± 8 HV, respectively. A slightly higher microhardness value measured for HT1 can be attributed to the presence of the needle-like δ phase observed on the cell and grain boundaries. The microstructure of the other three samples consists of the strengthening γ′, and γ″ precipitates and MC carbides with different size distributions. Despite the observed differences in the size distribution of the γ′ and γ″ precipitates, no significant variation in the microhardness value was observed for the HT2, HT3, and HT4 conditions.

#### 3.4.2. Tensile Properties

**Figure 14:**
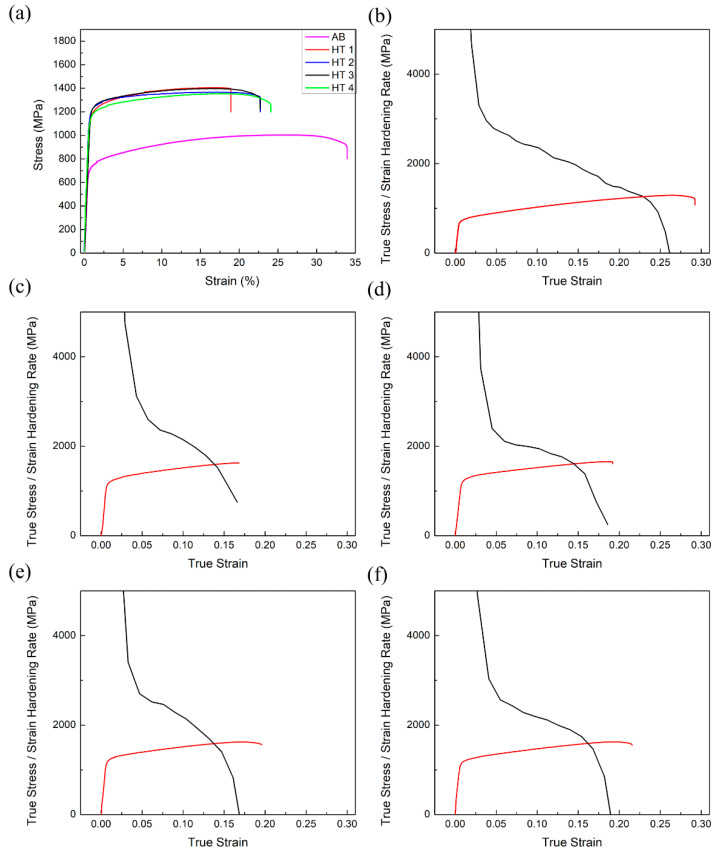
Representative (**a**) engineering stress-strain curves of the IN718 specimens in different conditions, true stress and strain hardening rate vs. true strain of the (**b**) AB, (**c**) HT1, (**d**), HT2, (**e**) HT3, and (**f**) HT4 specimens.

Uniaxial tensile tests were conducted to characterize the room temperature mechanical behavior of the in envelope additive (LPBF)/subtractive manufactured IN718 specimens. Representative engineering stress-strain curves of the AB and heat-treated specimens are shown in Figure 14a. The tensile properties of the specimens show distinct differences before and after being subjected to the heat treatments. A detailed summary of the mechanical property results for each condition in comparison with heat-treated (HT1) wrought IN718 [29] is also given in Table 5. The average YS, UTS, and elongation of the AB specimen were 790.8 ± 6.5 MPa, 1007.7 ± 5.8 MPa, and 34.0 ± 2.5%, respectively. When compared with standard wrought IN718 [29], lower YS (by 24%) and UTS (by 18%) values were observed in the AB condition. However, the elongation of the AB specimen was three times the elongation of the wrought IN718 [29]. In addition, since no change was observed in the porosity during the applied heat treatments, the difference in the mechanical properties can be directly associated with the microstructural changes. The lower strength and higher elongation of the AB specimen compared with the fully heat-treated wrought specimen can be associated with the absence of the strengthening γ′ and γ″ precipitates after LPBF fabrication. Relative to the AB specimen condition, each applied heat treatment resulted in an increase in the strength and a decrease in the elongation mainly due to the precipitation of the γ′ and γ″ precipitates. Despite the observed differences in their microstructures, all the heat-treated specimens showed statistically similar YS and UTS values that are 40–52% and 34–38% higher than the AB specimen, respectively. Compared to the AB condition, the applied heat treatments reduced the elongation to failure by 30–47%. The largest decrease in elongation (47%) was observed in the HT1 condition, while the HT4 yielded the best elongation of all the heat-treated specimens (24%). It is also worth noting that the observed YS, UTS, and elongation values for these specimens were considerably above the standard requirement for wrought IN718 [29].

For a better understanding of the room temperature plastic deformation behavior of the AB and heat-treated specimens, the true stress and strain hardening rate of each specimen were plotted as a function of the true strain. As shown in Figure 13, two stages of deformation could be identified for all the specimens before they reached the plastic instability region. During the first stage, the strain hardening rate quickly dropped at the onset of plastic deformation. Once plastic deformation started, the decrease in the strain hardening rate slowed down until necking. It should be noted that each specimen had a monotonic decrease in the strain hardening rate throughout the second stage of the deformation. The strain-hardening rate (∂σ/∂ε) then dropped below the true stress (σT), where plastic instability started according to Considere’s criterion [52]. The true strain at which the necking started was highest for the AB specimen at a value of 22.9 ± 0.7%. The calculated onset of necking was statistically similar for the HT1(14.4 ± 0.7%), HT2 (14.7 ± 0.2%), and HT3 (14.1 ± 0.9%) specimens and earlier compared to the AB and HT4 (16.4 ± 0.2%) conditions. It is noteworthy that the non-standard heat treatment (HT4) resulted in the longest range of strain hardening among all the heat treated conditions.

Considering the absence of precipitation after LPBF fabrication, the main effective strengthening mechanism in the AB specimen is dislocation strengthening provided by the cellular structure. It is reported that the rapid heating and cooling cycles observed in LPBF IN718 result in the formation of a heterogeneous dislocation structure consisting of high dislocation density cell walls with low dislocation density in the cell interiors [53]. The high dislocation density in the cell walls provides strengthening by hindering the dislocation motion. It has also been reported that this network dislocation structure provides stable plastic flow and higher elongation values at fracture [33]. After the specimens are subjected to heat treatments, this cellular substructure can dissolve, as evidenced by the microstructures of the HT1, HT2, and HT3 specimens. Hence, precipitation hardening is the predominant strengthening mechanism in these specimens. Generally, the gain in strength is directly correlated with the precipitate size [42]. However, in this study, despite the differences in the size distribution of the γ′ and γ″ precipitates, all three specimens showed similar strength values and necking behavior. The lower elongation at break observed for the HT1 specimen can be attributed to the presence of the needle-like δ phase. For wrought and cast alloys, the δ phase provides grain boundary pinning and is necessary to achieve fine grain size during forging [54]. On the other hand, for LPBF fabricated IN718, the δ phase is not required for grain size control and its dissolution is beneficial, as all the Nb is then available for γ′/γ″ precipitation [54]. Therefore, the presence of the δ phase in HT1 contributes to the grain boundary pinning during heat treatment but does not provide any additional strengthening. As shown in Figure 14c, the rate of decrease in the strain hardening rate is highest for the HT1 specimen and its slope remains almost the same after reaching the plastic instability region, which, in turn, results in the observed low elongation at break. Finally, the longest range of strain hardening observed in the HT4 specimen can be attributed to the combined strengthening contributions of the cellular sub-grain structure, extremely fine γ′/γ″ precipitates, and the fine carbide particles. The cellular structure observed in the AB specimen is preserved after HT4. This structure is reported to provide stability during plastic deformation as the dislocation slip can transfer across the cells, leading to an increase in strength without sacrificing elongation [33].

**Figure 15:**
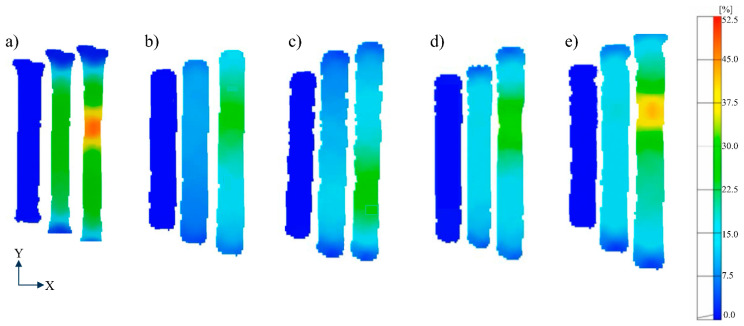
DIC strain maps (elastic, uniform deformation and just before fracture) captured during uniaxial tensile testing of IN718 specimens in (**a**) AB, (**b**) HT1, (**c**), HT2, (**d**) HT3, and (**e**) HT4 conditions, showing sequential images from elastic deformation, uniform plastic deformation stages and just before fracture.

Figure 15 illustrates the DIC strain maps that were recorded during uniaxial tensile testing of the IN718 specimens with different heat treatment conditions. Three snapshot images of the stress distribution for each specimen condition are presented, starting from the elastic deformation stage, going up to the uniform deformation stage, and then the stage just before fracture. The DIC strain maps for each condition showed that the strain at the uniform deformation stage was highest for the AB specimen (~30%), lowest for HT1 (~7%), and about 15% for HT2, HT3, and HT4. The homogeneous deformation behavior of the AB specimen over a longer strain range can be associated with the stable plastic deformation provided by the cellular sub-grain structure. Another difference observed was in their strain localization intensities at fracture. The AB specimen had the highest strain localization of ~52% at fracture. This may be attributed to the low strength and high elongation observed in this specimen. The strain maps for HT1, HT2, and HT3 showed similar local strain values of 27%, 32%, and 31% just before the fracture. This observation is in good agreement with the similar onset of necking for these three specimens according to Considere’s criterion, and the lowest elongation at break was observed for the HT1 specimen. The HT4 specimen had a local strain value of 40% just before fracture. Similar to the AB specimen, the higher range of homogeneous deformation and higher strain at fracture observed in this specimen can also be associated with the unique deformation behavior exerted by the hierarchical microstructure obtained from LPBF, which is retained after HT4 and provides a balanced performance with both high strength and high elongation.

### 3.5. Fractography

**Figure 16:**
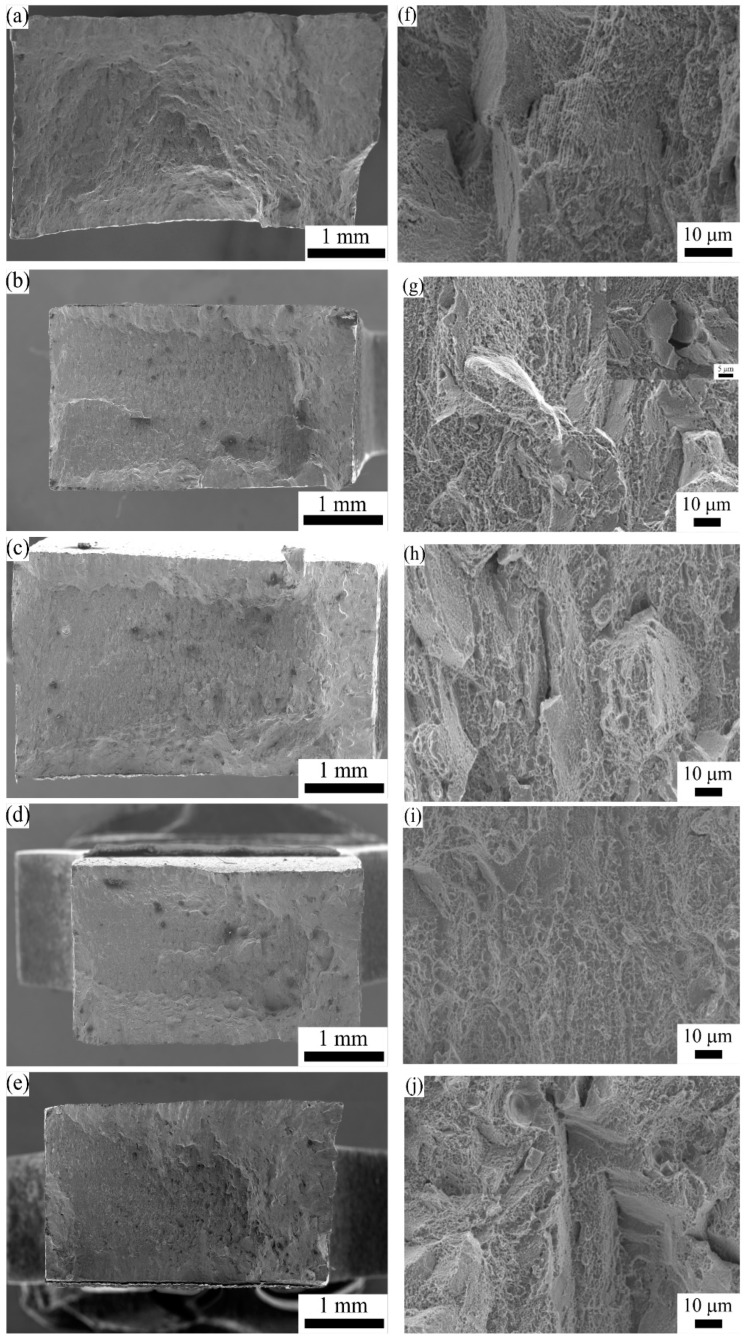
The tensile fracture surface of the IN 718 specimens in (**a**,**f**) AB, (**b**,**g**) HT1, (**c**,**h**) HT2, (**d**,**i**) HT3, and (**e**,**j**) HT4 conditions.

The fracture surfaces of each specimen after uniaxial tensile testing were analyzed to understand the effect of the heat treatment conditions on the fracture behavior. Representative SEM micrographs of each specimen condition are shown in Figure 16. No porosity or any other defects, such as unmelted powder or lack of fusion porosity, was observed on the fracture surfaces of any of the specimens. From the low magnification micrographs of all the tested specimens (Figure 16a–e) it is recognizable that the fractured surface of all the specimens consists of a central rupture zone with large dimples and a relatively flat area on the sides where fast crack propagation occurred. The morphology of the fracture surface proves that the fracture occurred mainly in a ductile manner following a transgranular path. This is in good agreement with the high elongation at fracture observed for all the specimens.

From the higher magnification fractography of the AB specimen, as illustrated in Figure 16f, a large number of dimple colonies homogeneously distributed along the fracture surface is clearly visible along with a small number of flat cleavage marks. Additionally, the cellular sub-grain structure is still visible on the fracture surface of the AB specimen, revealing that void formation started at the cell boundaries. These areas are where the presence of the Laves phase was observed, which points to the role of the Laves phase on crack initiation and propagation in the AB specimen. However, both the fracture surface analysis and mechanical property characterization show that the dominant fracture mechanism is ductile with high elongation and high local strains at fracture. The high magnification micrograph of the fracture surface of the HT1 specimen also shows the presence of dimple colonies distributed over the fracture area (Figure 16g). However, in this specimen, flat areas with small voids, approximately the same size as the δ needles, were observed as shown in the inset image. These voids suggest that decohesion between the δ needles and the matrix occurred during tensile deformation. Even though the discrete nature of the δ phase prevented the crack propagation along the boundaries, the lower elongation observed in this specimen can be associated with this (decohesion) phenomenon. The HT2, HT3, and HT4 specimens showed very similar fracture characteristics, as shown in Figure 16 h–j, respectively. Homogenous distribution of the dimple colonies is once again visible for all three specimens. Additionally, a few shallow voids having a similar size to the observed globular carbide particles are also visible on the fracture surfaces of each specimen. It is also worth noting that even though the cellular sub-grain structure was still observed in the HT4 specimen, the sub-grain boundaries did not act as crack initiation sites. The main reason behind this is the dissolution of the Laves phase during the short solutionizing stage in the HT4 heat treatment cycle.

## 4. Conclusions

This study investigated the microstructure and mechanical properties of IN718 after additive (LPBF)/subtractive manufacturing and four different standard and non-standard heat treatments. For this purpose, benchmarking coupons of IN718 were fabricated using an in envelope additive/subtractive hybrid manufacturing system. The coupons were then examined for surface finish both with and without high-speed micro-machining. The microstructure of the IN718 specimens was investigated thoroughly in the as-fabricated condition and following post-process heat treatments. Finally, the mechanical property results and fracture behaviors of IN718 in the different conditions were related to the processing effects on the microstructure. The following conclusions can be drawn from this study:

1. Additive/subtractive manufacturing results in a surface finish with lower roughness compared to the AB surface, allowing the use of a more flexible window for LPBF processing without consideration of the additive surface finish.
2. Parts without any defects having 99.6–99.9% relative density were obtained with the processing parameters optimized for high-efficiency fabrication. µCT analysis showed that the majority of the pores were spherical and smaller than 30 µm. The density and pore size distribution of the specimens was maintained following all the applied heat treatments.
3. The AB microstructure consisted of columnar grains parallel to the building direction, mainly aligned along the <100> direction. The texture, size, and aspect ratio of the grains were maintained after each of the applied heat treatments, showing that the grain morphology was stable at elevated temperatures.
4. The AB specimen exhibited a cellular dendritic sub-grain structure having fine discrete carbide particles and Laves phase on the boundaries without the presence of γ′/γ″ precipitates. This cellular sub-grain structure dissolved after the three standard heat treatments and was preserved only after HT4, a non-standard heat treatment adapted for additively processed IN718.
5. After the specimens were subjected to the HT1, the formation of the δ phase along with the γ′/γ″ precipitates was observed.
6. HT2 and HT3 each resulted in the formation of spherical carbide particles and γ′/γ″ precipitates. Hence, the extra solutionizing step in the HT2 did not affect the phases formed in the final microstructure. The main difference between the microstructure of these two specimen conditions was the size of the precipitates and carbide particles. The carbide particles were smaller in the HT3 condition due to the limited coarsening at lower homogenization temperature, whereas the size distribution of the strengthening precipitates was larger due to the higher aging temperature of this specimen.
7. The non-standard heat treatment with a short solutionizing step and one-step aging (HT4) was effective for Laves phase dissolution, while preserving the carbide particles. The HT4 condition also had the finest γ′/γ″ size distribution.
8. The AB specimen had the lowest strength and hardness and the highest elongation. The strength and hardness were improved, while the elongation decreased after each of the applied heat treatments due to the precipitation of the strengthening phases. The strength and elongation of all heat-treated specimens were above the minimum requirement for wrought IN718 in the heat-treated condition. Despite the differences observed in their microstructures, all heat-treated specimens showed similar strength values. HT1 resulted in the lowest elongation and the highest rate of softening due to the presence of the δ phase. The cellular sub-grain structure observed in the HT4 specimen resulted in the highest elongation at break, without the strength-ductility trade-off.

## Figures and Tables

**Table 1:** Chemical composition of the starting IN718 powder feedstock.

Element	Cr	Mo	Nb	Al	Ti	C	Si	Mn	Ni	Fe
Wt.%	19.19	3.07	5.25	0.64	0.93	0.04	0.15	0.14	52.37	18.22

**Table 2:** Summary of the specimen conditions and applied heat treatments.

Designation	Standard	Treatment	Temperature (°C)	Time (h)	Cooling
AB	-	-	-	-	-
HT1	AMS 5663 [30]	Solution	980	1	AC
		Age	720	8	FC to 620 °C (55 °C/h)
			620	8	AC
HT2	AMS 5383 [31]	Homogenize	1080	1.5	AC
		Solution	980	1	AC
		Age	720	8	FC to 620 °C
			620	8	AC
HT3	AMS 5664 [32]	Homogenize	1065	1	AC
		Age	750	10	FC to 620 °C
			650	8	AC
HT4	Non-standard [33]	Solution	1020	0.25	WQ
		Age	720	24	AC

AC = Air Cooled; FC = Furnace Cooled; WQ = Water quenched.

**Table 3:** Measured linear and areal roughness values from the vertical as-built and machined surfaces of IN718 specimens.

Condition	Linear Roughness (µm)		Areal Roughness (µm)	
	Ra	Rz	Sa	Sz
As-built	5.24 ± 0.37	30.94 ± 1.70	9.17	66.52
Machined	0.66 ± 0.06	4.05 ± 0.53	1.43	13.90

**Table 4:** Archimedean density, overall volume, and the number of detected voids from the µ-CT of IN718 specimens in each condition.

Condition	Archimedean Density (g/cm^3^)	Total Pore Volume (%)	Pore Density (#/mm^3^)
AB	8.16 ± 0.01	0.006	32
HT1	8.18 ± 0.02	0.003	24
HT2	8.18 ± 0.01	0.005	29
HT3	8.18 ± 0.03	0.008	37
HT4	8.18 ± 0.02	0.007	32

**Table 5:** Summary of the room temperature tensile properties of IN718 specimens in different conditions.

CONDITION	YS (MPA)	UTS (MPA)	ELONGATION (%)
AB	790.8 ± 6.5	1007.7 ± 5.8	34.0 ± 2.5
HT1	1133.9 ± 31.6	1387.2 ± 18.5	18.3 ± 0.7
HT2	1204.6 ± 7.0	1381.6 ± 11.8	21.4 ± 1.3
HT3	1189.5 ± 12.3	1387.1 ± 10.2	21.0 ± 2.1
HT4	1102.7 ± 28.9	1346.7 ± 22.6	24.0 ± 0.1
Wrought [29]	1034	1241	12

## Data Availability

Not applicable.
